# Supplementary material for: Three-Dimensional Ultraviolet Fluorescence Imaging in Cultural Heritage: A Review of Applications in Multi-Material Artworks
Source: J Imaging. 2025 Jul 21;11(7):245. doi: 10.3390/jimaging11070245 (PMC12295401; doi:10.3390/jimaging11070245)
Supplement: Supplementary file 1 [file jimaging-11-00245-s001.zip › jimaging-3696222-supplementary.pdf]

Journal of Imaging

Supplementary Materials for the Review paper

Three-dimensional ultraviolet fluorescence imaging in cultural heritage: a review of applications in multi-material artworks

Luca Lanteri [1], Claudia Pelosi [1,*] and Paola Pogliani[2]

[1] Department of Economics, Engineering, Society and Business Organization, University of Tuscia, Largo dell'Università, 01100 Viterbo, Italy; llanteri@unitus.it (LL)
[2] Department for Innovation in Biological, Agro-Food and Forest Systems, University of Tuscia, Largo dell'Università, 01100 Viterbo, Italy; pogliani@unitus.it (PP)

* Correspondence: pelosi@unitus.it (CP)

Table S1. Sketchfab links to the 3D UVF digital models and the publication details.

**Link to the 3D UVF model**	**Publication**
https://skfb.ly/6EMy7	Lanteri et al. [33]
https://sketchfab.com/3d-models/san-icodono-ridotto-623eeb3a3e6c40888f0c9663c5fc4adf	Lanteri and Pelosi [42]
https://sketchfab.com/3d-models/san-rodonio-tempo-1-187ef9beb2de45388bde63390591a778	Lanteri and Pelosi [42]
https://sketchfab.com/3d-models/san-filomelo-3ef74f8fe0af4d2291b6898d6f72d391	Lanteri and Pelosi [42]
https://sketchfab.com/3d-models/san-leonardo-7bcff9ed23ae4221a50ba52b06285679	Lanteri and Pelosi [42]
https://sketchfab.com/3d-models/santa-rosalia-8b4cf6c05837433c8c383b6266bf46fb	Lanteri and Pelosi [42]
https://sketchfab.com/3d-models/santo-stefano-ac0f26ccea1d466a822a499df5df1d3b	Lanteri and Pelosi [42]
https://sketchfab.com/3d-models/pio-v-3d-uvf-model-063c014bb7734581b3ebfca95d1ab85e	Lanteri and Pelosi [12]
https://sketchfab.com/3d-models/copricapo-uvf-45e43bcc474b470e8c8b876db917ba3e	Colantonio et al. [39]
https://sketchfab.com/3d-models/tomba-degli-scudi-tar-quinia-7a183dcbe1e84bb199babcc0b30c3905	Rinaldi et al. [86]
https://sketchfab.com/3d-models/modello-3d-bambinello-corona-8d6a084e53144fb98174b8a0671ca4d5	Ceci et al. [41]
https://sketchfab.com/3d-models/madonna-del-carmine-3d-model-672bf63e67f44f40b102dd789658bb0a	Published on Sketchfab
https://sketchfab.com/3d-models/sant-andrea-3d-uvf-5f10ae5fb00242e293aaa297473dad68	Published on Sketchfab

For the references see the main document
